# Case of Abortion Followed by Septic Infection from Retained Placenta

**Published:** 1878-04

**Authors:** 


A Case of Abortion Followed by Septic Infection from Retained Placenta; Its Successful Treatment.—Sunday afternoon, at 3 o'clock, November 4, 1877, was called to see Mrs. W——, four months gone in pregnancy. Found her suffering with periodical pains. She stated to me that she was flowing freely. I immediately made an examination per vaginum and my finger came in contact with a partially expelled fœtus. The patient was flooding terribly, and after some difficulty I succeeded in getting hold of the fœtus and extracted it at once. I then cut the cord and practiced "Crede's method," with a view to stimulate contractions and expel the placenta, which seemed firmly adherent. After manipulating for a short time, without success, and the flooding continuing, I introduced a sponge tampon up to the os. This completely checked the hemorrhage. I then gave appropriate doses of fluid extract of ergot at stated hours during the night, and let the tampon remain until morning, hoping from the stimulating action of its presence against the os and the effect of the ergot, to find the placenta expelled entire when it was removed, but in this I was disappointed.

November 5th.—Called and found the patient complaining of abdominal pain and tenderness, with some acceleration of the pulse. These symptoms, with some others, began to make me suspect septic infection. I removed the tampon and found the hemorrhage checked and the placenta about one-third expelled from the os. Seizing it with my fingers I tried to extract it, but my efforts were of no avail, it was firmly attached.

The discharge was so very offensive, and the symptoms so strong of septic poison, that after two or three attempts in this way, and being so anxious to relieve the woman and get rid of the decomposing placenta, I introduced a Sim's speculum, and got hold of the presenting part, and with a pair of close-shutting tooth forceps—not having placental forceps at hand—succeeded in extracting about one-half of it. Her bowels having been bound up for several days, and feeling very uncomfortable, I ordered castor oil followed by opiates to relieve pain, with fomentations, and also continued the ergot.

November 6.—Called and found the patient suffering with severe pain and soreness of the bowels. Bowels had moved; had neglected to take the opium, tympanitic condition, considerable fever, pulse full and strong, anxious countenance, with very offensive discharge. Examination revealed a portion of the placenta in the os. Being unable to remove it, and the condition growing desperate, I remembered of reading of injections of hot water being an efficacious remedy in such cases, and concluded to try it. I placed an oilcloth on the bed, drew the patient to the edge, laying her on her left side with head and shoulders somewhat elevated, then injected a steady stream of water as hot as the patient could bear it against the os for several minutes. This caused contraction and the placenta was expelled. I would here state that moving the patient caused intense pain, but after using the injections for a few moments the pain ceased, and the patient expressed a degree of comfort. Ordered opiates and fomentations.

November 5th.—Found patient complaining of pain in the bowels; neglected to take the opium. General abdominal tenderness and tympanitic condition; pulse weak and quick; says she did not rest well the night before. More or less thirst, and anxious countenance. Ordered opiates and quinine, and turpentine stupes to abdomen, with vaginal injections of hot water to remove offensive discharge.

Evening.—Patient more comfortable, less soreness and tympanitis, pulse more natural. Continued vaginal injections three times a day with fomentation and opiates.

November 8th.—Patient feeling considerable better, less pain, tympanitic condition passing off, some pain and soreness on moderate pressure, less discharge, complains of breast paining her, rested well, some headache, bids fair for recovery. Continued treatment as before.

November 9th.—Patient improving, and from this time on has continued to improve and is now up and around. This shows the value of opium in the treatment of this dangerous disease, also the value of hot water injections in removing the placenta, offensive discharge, etc., thereby removing the focus of septic infection. These remedies should never be neglected. I consider the injections just as essensial to successful treatment as the opium in those cases.—*Ohio Medical Recorder.*

